# Electrocatalytic CO_2_ Reduction Empowered by 2D Hexagonal Transition Metal Borides

**DOI:** 10.1002/advs.202500977

**Published:** 2025-04-01

**Authors:** Yaxin Di, Zhiqi Wang, Guangqiu Wang, Junjie Wang

**Affiliations:** ^1^ State Key Laboratory of Solidification Processing School of Materials Science and Engineering Northwestern Polytechnical University Xi'an Shaanxi 710072 P. R. China

**Keywords:** electrochemical CO_2_ reduction reaction, first principles, functional groups, *h*‐MBenes

## Abstract

Electrocatalysis holds immense promise for producing high‐value chemicals and fuels through the carbon dioxide reduction reaction (CO_2_RR), advancing global sustainability and carbon neutrality. However, conventional electrocatalysts based on transition metals are often limited by significant overpotentials. Since the discovery of the first hexagonal MAB (*h*‐MAB) phase, Ti_2_InB_2_, and its 2D derivative in 2019, 2D hexagonal transition metal borides (*h*‐MBenes) have emerged as promising candidates for various electrochemical applications. This study presents the first theoretical investigation into the CO_2_RR catalytic properties of pristine *h*‐MBenes (*h*‐MB) and their ─O (*h*‐MBO) and ─OH (*h*‐MBOH) terminated counterparts, focusing on metals such as Sc, Ti, V, Zr, Nb, Hf, and Ta. These results reveal while *h*‐MB and *h*‐MBO exhibit poor catalytic performance due to overly strong or weak interactions with CO_2_, *h*‐MBOH shows great promise. Notably, ScBOH, TiBOH, and ZrBOH display exceptionally low limiting potentials (*U*_L_) of −0.46, −0.53, and −0.64 V, respectively. These findings uncover the unique role of ─OH in tuning the electronic properties of *h*‐MBenes, thereby optimizing intermediate adsorption, which prevents excessive binding and enhances catalytic efficiency. This research offers valuable insights into the potential of *h*‐MBenes as highly efficient CO_2_RR catalysts, underscoring their versatility and significant prospects for electrochemical applications.

## Graphical abstract

This work used DFT calculations to discover a series of *h*‐MBenes with extraordinary eCO_2_RR catalytic activity and selectivity, revealing the mechanism of functional groups regulating eCO_2_RR performance. This study highlights the potential of *h*‐MBenes as highly efficient eCO_2_RR catalysts, shedding light on their versatility and significant prospects for electrochemical applications.

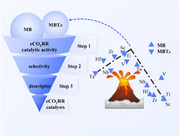

## 1 Introduction

In 2023, the concentration of carbon dioxide (CO_2_) in the atmosphere, primarily from fossil fuels, reached a new high of 421.08 parts per million (ppm).^[^ ^1^ ^]^ This increase is expected to exacerbate environmental issues such as severe climate change, rising sea levels, and ocean acidification, potentially threatening both the survival and sustainable development of humanity.^[^ ^2^ ^]^ Addressing these concerns requires effective strategies for mitigating CO_2_ emissions. One promising approach is converting CO_2_ into high‐value chemicals and fuels through various processes: thermochemical,^[^ ^3^ ^]^ photochemical,^[^ ^4^ ^]^ electrochemical,^[^ ^5^ ^]^ or biological.^[^ ^6^ ^]^ Among these, electrochemical CO_2_ reduction reaction (eCO_2_RR) has attracted significant interest due to its potential economic benefits.

The eCO_2_RR process offers precise control over the production of high‐value chemicals and fuels by adjusting electrochemical parameters and selecting suitable catalysts, all achievable under mild conditions of room temperature and pressure. This method facilitates the generation of products with varying carbon chain lengths, encompassing C_1_, C_2_, and C_3_ chemicals. C_1_ chemicals include carbon monoxide (CO),^[^ ^7^ ^]^ methane (CH_4_), formic acid (HCOOH),^[^ ^8^ ^]^ and methanol (CH_3_OH);^[^ ^9^ ^]^ C_2_ chemicals comprise ethylene (C_2_H_4_),^[^ ^10^ ^]^ acetic acid (CH_3_COOH), and ethanol (CH_3_CH_2_OH);^[^ ^11^ ^]^ and C_3_ chemicals consist of acetone (H_3_CCOCH_3_)^[^ ^12^ ^]^ and propanol (CH_3_CH_2_CH_2_OH).^[^ ^13^ ^]^

These chemicals can function as innovative renewable energy carriers or raw materials, promoting the natural carbon cycle and significantly advancing sustainable development. Among these, C_1_ products stand out in particular. CO serves as a crucial raw material in the production of fuels through Fischer‐Tropsch synthesis.^[^ ^14^ ^]^ HCOOH is utilized across diverse applications, including leather tanning, pharmaceuticals, and electroplating, demonstrating substantial market potential.^[^ ^15^ ^]^ CH_3_OH is a vital precursor in the chemical industry, acting as a clean fuel and a key component in the production of chemicals such as silicones and plastics.^[^ ^16^ ^]^ Lastly, CH_4_, the primary constituent of natural gas, is an essential raw material for synthesizing various chemical products, including aromatic hydrocarbons.^[^ ^17^ ^]^

However, the inert nature of the C═O bond in CO_2_ molecules, with a dissociation energy of up to 750 kJ mol^−1^, necessitates the full activation of CO_2_ as a prerequisite for eCO_2_RR. Furthermore, eCO_2_RR requires a proton‐coupled multi‐electron transfer process, typically involving 2 to 12 electron transfers, to generate various end products. Consequently, eCO_2_RR involves diverse reaction pathways and intricate mechanisms. Furthermore, it is important to highlight that the hydrogen evolution reaction (HER), a 2‐electron reaction, is more favorable kinetically and thermodynamically compared to eCO_2_RR. When attempting to enhance CO_2_ reduction by increasing the electrode voltage, HER as a side reaction is inevitably promoted, leading to low Faraday efficiency. Therefore, it is essential to identify eCO_2_RR electrocatalysts with high catalytic activity and favorable selectivity to address these challenges.

2D materials, including *g*‐C_3_N_4_,^[^ ^18^ ^]^ hexagonal boron nitride (*h*‐BN),^[^ ^19^ ^]^ metal‐organic framework materials (MOF),^[^ ^20^ ^]^ transition metal carbides/nitrides (MXenes)^[^ ^21^ ^]^ and borides (MBenes),^[^ ^22^ ^]^ exhibit unique properties that position them as promising electrocatalysts. These materials are characterized by a high specific surface area, specific strength, elevated surface activity, and tunable electronic structures. In particular, hexagonal MBenes (*h*‐MBenes)^[^ ^23^ ^]^ have garnered increasing attention following the discovery of the first hexagonal layered transitional metal boride (*h*‐MAB), Ti_2_InB_2_, in 2019. Subsequent theoretical studies have revealed that *h*‐MAB phases constitute a diverse family of materials with over 130 potential members.^[^ ^24^ ^]^ The A‐layers of these *h*‐MAB phases can be exfoliated to create a series of 2D *h*‐MBene materials.^[^ ^23^, ^25^ ^]^ Both experimental and theoretical investigations have highlighted the electrochemical applications of *h*‐MBenes such as Hf_2_BO_2_ and Mo_2_ErB_3_T_2.5_ (T = O, F, and Cl), which show promise as a catalyst for hydrogen evolution reactions (HER),^[^ ^26^ ^]^ W_8/9_Cu_1/9_B, which is promising electrocatalyst for nitrogen reduction reaction (NRR),^[^ ^27^ ^]^ and HfBO, which is considered a potential anode material for lithium‐ion batteries.^[^ ^28^ ^]^

The catalytic activity of *h*‐MBenes arises from the diverse transition metals exposed on their surfaces, which act as catalytic active sites. Additionally, the hexameric ring formed by B─B bonds imparts stability under catalytic conditions, granting *h*‐MBenes exceptional characteristics that are comparable to traditional metal catalysts used in eCO_2_RR. Beyond their intrinsic properties, *h*‐MBenes can be further enhanced by the introduction of functional groups (e.g., ─F, ─O, ─OH), which can effectively modify their catalytic behavior.^[^ ^29^ ^]^ This approach provides a promising avenue for designing eCO_2_RR catalysts with improved activity and selectivity.^[^ ^30^ ^]^ However, there is a notable gap in comprehensive research regarding the catalytic mechanisms of eCO_2_RR in *h*‐MBenes, particularly those terminated with surface functional groups. Consequently, further investigations are essential to elucidate the eCO_2_RR catalytic mechanisms of *h*‐MBenes, both with and without these modifications. Such studies will contribute significantly to advancing the understanding and application of *h*‐MBenes in sustainable catalysis.

**Figure 1:**
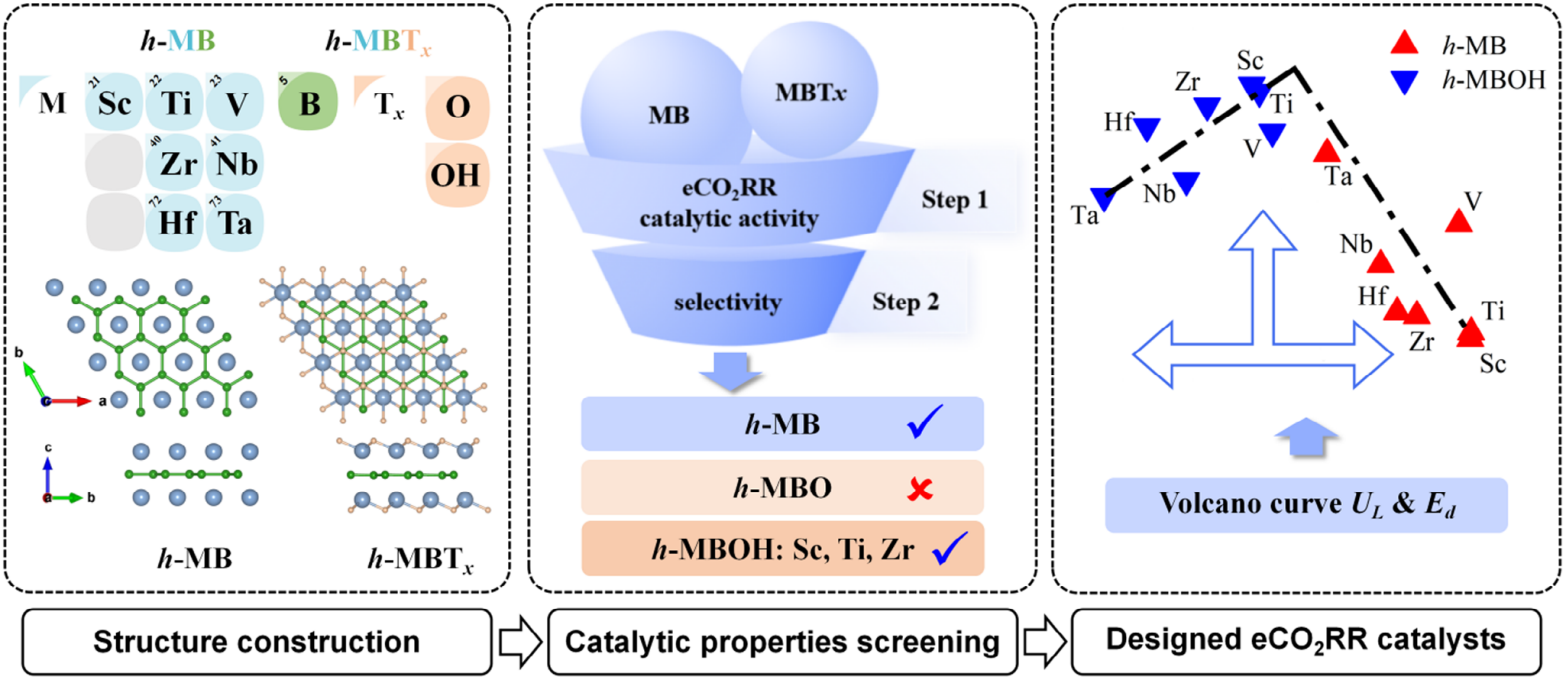
Schematic diagram illustrating the workflow of the study. It outlines the steps of structure construction, screening of eCO_2_RR catalytic properties, and the design of functionalized *h*‐MBs catalysts. In the left panel, blue, green, and brown spheres represent the metal (M) atoms, boron (B) atoms, and functional groups (T), respectively.

As shown in **Figure** **1**, this study investigates the electrochemical CO_2_ reduction reaction (eCO_2_RR) performance of stable pristine, ─O, and ─OH terminated MB‐type hexagonal MBenes (*h*‐MBs), which we previously screened as foundational structures for *h*‐MBenes.^[^ ^24^, ^28^ ^]^ Our primary focus is to evaluate their potential for converting CO_2_ into C_1_ products such as CH_4_, HCOOH, and CH_3_OH. This represents the first exploration of these materials in the context of eCO_2_RR. The rationale behind the selection of transition metals and functional groups is showed in the left panel of Figure 1. It should be pointed out that the current calculations are based on ideal crystal structures, without considering the potential defects (such as vacancies, edge sites) or stress‐induced structural distortions that may exist in actual preparation. These factors may have an impact on the catalytic properties of materials. Subsequent research will further explore this issue by combining experimental characterization and multi‐scale modeling.

Our findings reveal that functionalizing *h*‐MBs with ─OH groups significantly enhances their eCO_2_RR catalytic activity, whereas ─O functionalization is detrimental to their catalytic properties. Catalytic performance and selectivity analyses identify ScBOH, TiBOH, and ZrBOH as promising candidates, with limiting potentials of −0.46, −0.53, and −0.64 V, respectively, as shown in the central panel of Figure 1. Furthermore, we explored the electronic structures of these *h*‐MBene materials and found that their eCO_2_RR catalytic activity is influenced by the strength of CO_2_ activation, which correlates with the energy distribution of the *d*‐orbitals in *h*‐MBs.

Based on our theoretical results, we established a volcano‐type relationship between the eCO_2_RR limiting potential (*U*_L_) and the *d*‐band center (*E*_d_) for both 2D *h*‐MB and *h*‐MBOH structures, as presented in the right panel of Figure 1. Finally, a microkinetic analysis of the eCO_2_RR catalytic process for *h*‐ScBOH indicates that HCOOH is produced at a higher rate than CH_4_ and CH_3_OH under the studied conditions (pH 0 to 7, *U*_SHE_ = 0 to −1), suggesting that HCOOH is the primary product. These results provide valuable insights into the durability and stability of these catalysts throughout the eCO_2_RR process.

## 2 Results and Discussion

### 2.1 The eCO_2_RR Performance of 2D *h*‐MBs

In this study, we conducted a comprehensive investigation of the eCO_2_RR catalytic activity of 2D *h*‐MBs. The C═O bond energy in CO_2_ can reach up to 750 kJ mol^−1^, making effective activation a critical challenge for successful eCO_2_RR. To address this, we systematically explored *h*‐MBs with various metal components (Sc, Ti, V, Zr, Nb, Hf, and Ta) and surface functional groups (pristine, ─O, and ─OH). The choice of these transition metals was primarily based on their unfilled *d* orbitals (e.g., *3d^1^* for Sc, *3d^2^* for Ti), which facilitate CO_2_ adsorption and activation through *d‐π* interactions. Furthermore, the ─O and ─OH functional groups were selected for their ability to modulate the surface charge, electronic structure, and intermediate adsorption strength, allowing us to fine‐tune the catalytic performance.

Initially, we systematically examined the adsorption of CO_2_ on the seven *h*‐MBs. As shown in Figure 1a, the surface characteristics of the *h*‐MBs reveal three distinct adsorption sites for CO_2_: bridge, top, and hollow sites. Figure S1 (Supporting Information) illustrates the most stable adsorption configurations of CO_2_, which can be categorized into two types. In Type I (M = Ta), the O atoms form bonds with Ta atoms at the top site, while the C atom tends to absorb at the bridge site between two Ta atoms, resulting in a vertical V‐shape. Conversely, in Type II (M = Sc, Ti, V, Zr, Nb, and Hf), the interaction sites for C and O atoms with the M atoms differ from those in Type I; here, the atoms preferentially adsorb at the hollow site, creating a horizontal V‐shape. The V‐shaped bond angles (∠OCO) in both adsorption states are less than 180.0°: 132.4° for Type I and ≈115.0° for Type II. Subsequently, we calculated the adsorption energy of CO_2_ on the surfaces of the *h*‐MBs. The results indicate that the adsorption energies for CO_2_ on these surfaces range from −3.12 eV (for ScB) to −1.44 eV (for TaB), specifically: −3.12 eV (Sc), −3.27 eV (Ti), −2.20 eV (V), −2.69 eV (Zr), −1.75 eV (Nb), −2.61 eV (Hf), and −1.44 eV (Ta). These values demonstrate a stronger interaction between CO_2_ and *h*‐MBs compared to the adsorption energies on MXenes, which range from −2.31/−1.65 eV (TaN/HfC) to −0.70 eV (VN/NbC).^[^ ^31^ ^]^ Furthermore, the negative adsorption energies suggest that the chemisorption of CO_2_ on the surfaces of *h*‐MBs is a spontaneous process.

To analyze the changes in bond lengths of CO_2_ following adsorption, we calculated the average length values of the two C─O bonds in CO_2_ across seven *h*‐MBs. The results are presented in Figure S2a (Supporting Information). Notably, the C─O bonds of the adsorbed CO_2_ on *h*‐MBs exhibit significant stretching compared to those in the gaseous state (1.17 Å), with lengths ranging from 1.28 Å (TaB) to 1.40 Å (VB). In particular, the C─O bond lengths associated with type II adsorption are consistently ≈1.40 Å, reflecting a more pronounced increase compared to type I adsorption. Furthermore, we conducted a Bader charge analysis to evaluate the charge transfer from the *h*‐MBs surface to the CO_2_ molecule. The Bader charge calculations reveal a charge transfer ranging from 1.18 e (TaB) to 1.95 e (ScB), which further supports the complete activation of CO_2_ (Figure S2b, Supporting Information). These findings provide compelling evidence that *h*‐MBs effectively activate CO_2_, as indicated by the favorable and stable adsorption configurations observed.

**Figure 2:**
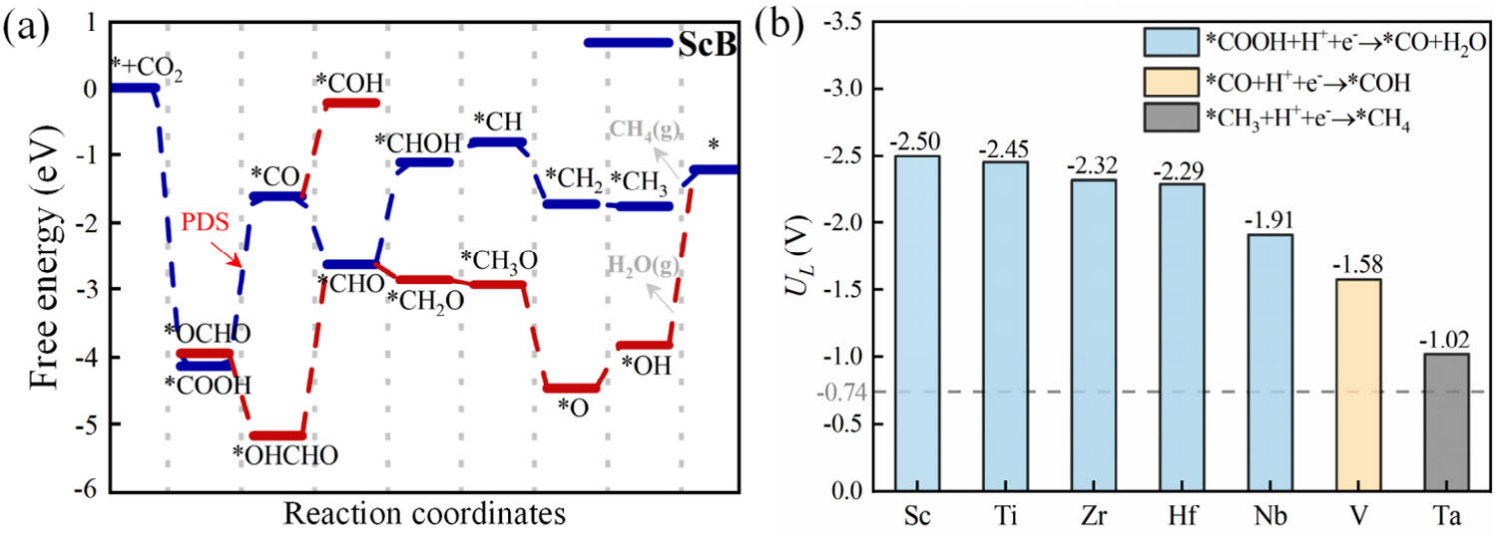
The eCO_2_RR catalytic activity of 2D *h*‐MBs. a) Gibbs free energy diagrams for the eCO_2_RR pathway on the ScB surface. b) Limiting potential and potential‐determined steps for eCO_2_RR on *h*‐MBs. In panel (a), the blue and red lines indicate favorable and unfavorable CO_2_ reduction pathways, respectively.

We further calculated the reaction pathways for the electrocatalytic reduction of CO_2_ to C_1_ products on seven *h*‐MBs to assess their catalytic activity. Using ScB as an example, we analyzed the Gibbs free energy changes for various intermediates involved in potential reduction pathways, as illustrated in **Figure** **2**a. The initial hydrogenation step can lead to the formation of ^*^OCHO, with an energy change of −3.93 eV, while ^*^COOH shows a larger energy change of −4.11 eV. This indicates that CO_2_ preferentially hydrogenates to ^*^COOH in the early stage.

Subsequently, ^*^COOH can dehydrate to produce ^*^CO and H_2_O, with an Δ*G* of 2.50 eV. Given the adsorption energy of CO on ScB is −2.22 eV, desorption is challenging, prompting further hydrogenation of CO. The intermediates ^*^COH and ^*^CHO depicted in Figure 2a reveal that the proton preferentially binds to the C atom of ^*^CO to form ^*^CHO (Δ*G* = −0.99 eV), rather than to the O atoms to form ^*^COH (Δ*G* = 1.38 eV). Consequently, the most energetically favorable pathway is ^*^CHO → ^*^CHOH → ^*^CH → ^*^CH_2_ → ^*^CH_3_ → CH_4_ (g), with energy barriers of 1.50, 0.31, −0.92, −0.03, and 0.54 eV, respectively. The adsorption energy of CH_4_ on ScB is 0.32 eV, facilitating its desorption from the surface. The limiting potential (*U*_L_) for this reaction pathway is −2.50 V, where the second hydrogenation step, ^*^COOH → ^*^CO, is identified as the potential‐determining step with a Δ*G*_max_ of 2.50 eV.

Additionally, we explored an alternative pathway (represented by red lines in Figure 2a), where the proton sequentially binds to the C atom in ^*^CHO until CH_4_ is generated and detached, followed by the reduction of O atoms to produce H_2_O, represented as ^*^CHO → ^*^CH_2_O → ^*^CH_3_O → ^*^O → ^*^OH → H_2_O (g). Although the fourth hydrogenation step to generate ^*^CH_2_O (Δ*G* = −0.22 eV) is thermodynamically more favorable than forming ^*^CHOH (Δ*G* = 1.50 eV), the final step of this pathway, ^*^OH → H_2_O (g) (Δ*G* = 2.59 eV), becomes the PDS, resulting in a slightly higher limiting potential (*U*_L_ = −2.59 V) compared to the previous pathway (*U*_L_ = −2.50 V). Therefore, this alternative pathway is less likely to occur.

Furthermore, Figure S3 (Supporting Information) illustrates the potential eCO_2_RR pathways for CO_2_ reduction on the other six *h*‐MBs (M = Ti, V, Zr, Nb, Hf, and Ta). Figure 2b summarizes the PDS and corresponding *U*_L_ values for these *h*‐MBs. For most *h*‐MBs (M = Ti, Zr, Nb, and Hf), the favorable eCO_2_RR pathway mirrors that of ScB: CO_2_ → ^*^COOH → ^*^CO + H_2_O → ^*^CHO → ^*^CHOH → ^*^CH → ^*^CH_2_ → ^*^CH_3_ → CH_4_ (g), with the PDS being ^*^COOH → ^*^CO + H_2_O (Figure 2b). In contrast, for TaB and VB, the preferred pathway is CO_2_ → ^*^COOH → ^*^CO + H_2_O → ^*^COH → ^*^C → ^*^CH → ^*^CH_2_ → ^*^CH_3_ → CH_4_ (g), with their respective PDS being ^*^CH_3_ → CH_4_ (g) for TaB and ^*^CO → ^*^COH for VB (Figure 2b). Among these *h*‐MBs, TaB exhibits the lowest limiting potential of −1.02 V, while ScB has the highest at −2.50 V. Notably, all *h*‐MBs possess higher limiting potentials than the conventional Cu catalyst (−0.74 V), which contributes to their limited catalytic activity.

### 2.2 The Stability and eCO_2_RR Performance of 2D *h*‐MBO and *h*‐MBOH

Due to the high surface activity of 2D non‐van der Waals materials, *h*‐MBs are often terminated by functional groups such as ─O and ─OH.^[^ ^29^ ^]^ The presence of these functional groups significantly influences the electronic structure of MBenes, thereby affecting their electrocatalytic properties. The surface functional state of *h*‐MBenes can be transformed by external environmental factors such as pH and applied voltage. The transformation occurs in the following manner:

(1) $$-O+H^{+}+e^{-}↔-\mathrm{OH}$$

(2) $$-\mathrm{OH}+H^{+}+e^{-}↔\mathrm{slab}+H_{2}O$$

To examine the functional state of *h*‐MBenes under various applied potentials, the redox potential at pH = 0 was calculated for *h*‐MBenes with 1 monolayer (ML) of ─OH, 1 ML of ─O, and 1/2 ML of ─OH & 1/2 ML of ─O. Additionally, to extend the pH range from 0 to 14, surface Pourbaix diagrams were calculated using the following formula:

(3) $$U_{\mathrm{SHE}}=U_{\mathrm{RHE}}\;-\frac{k_{B}T\times pH}{e}\;\ln\left(10\right)=U_{\mathrm{RHE}}\;-0.059\times pH$$

**Figure 3:**
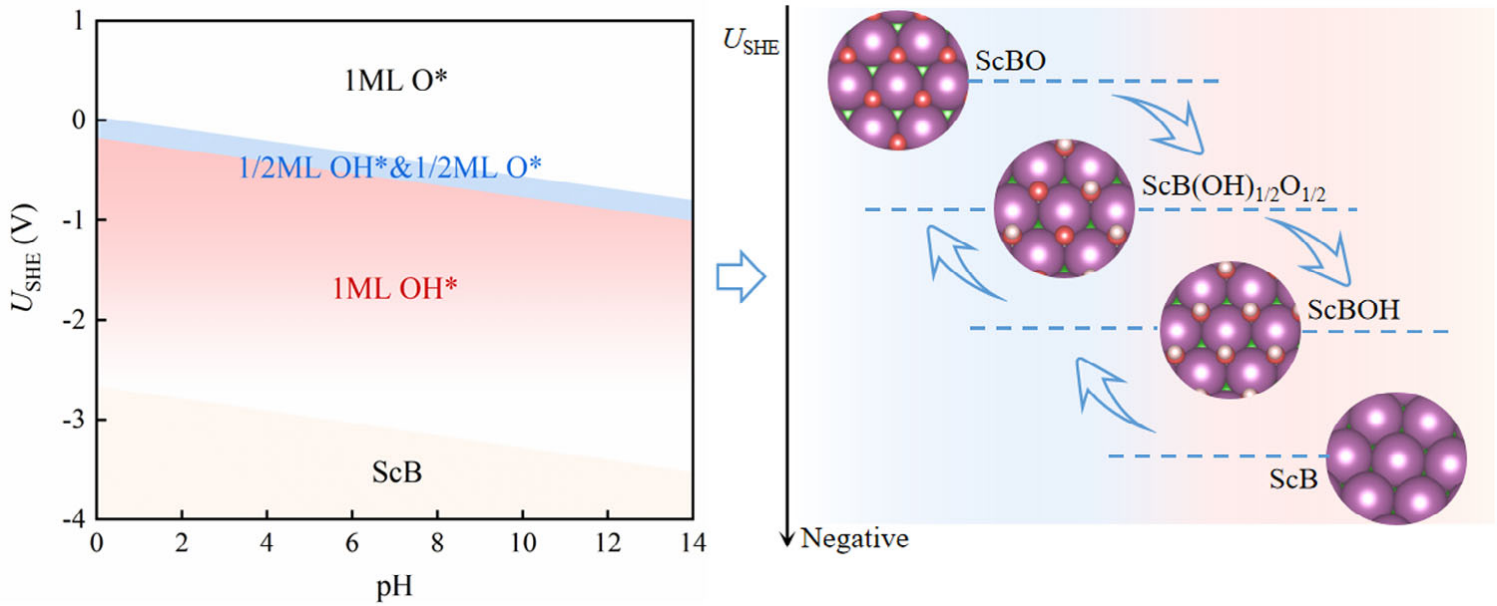
Stability of *h*‐ScBT*_x_* (T = O or OH, 0 ≤ *x* ≤ 1). Calculated surface Pourbaix diagram (left panel) and illustration of surface functional groups (right panel) for *h*‐ScBT*_x_* (T = O or OH, 0 ≤ *x* ≤ 1).

**Figure** **3** presents the calculated Pourbaix diagram for ScB as an example. The Pourbaix diagrams for other *h*‐MBs (M = Ti, V, Zr, Nb, Hf, and Ta) are provided in Figure S4 (Supporting Information). As the cathode voltage increases, the ─O functional groups on the surface of *h*‐MBenes undergo a sequential reduction process. Initially, ─O groups are reduced to ─OH, and at more negative voltages, they eventually transform into H_2_O and desorb from the surface. In a strongly acidic solution (pH = 0), at a voltage of 0.04 V, half of the ─O groups of ScBO are reduced to ─OH, and at −0.18 V, all ─O groups are fully converted to ─OH (Figure 3). At a voltage of −2.68 V, all ─OH groups are reduced to H_2_O, exposing the underlying Sc metal atoms on the surface. A similar phenomenon is observed for the other *h*‐MBTs (Figure S4, Supporting Information). This indicates that these *h*‐MBT*_x_* materials (M = Sc, Ti, V, Zr, Nb, Hf, and Ta; T = O or OH; 0 ≤ *x* ≤ 1) can remain stable within specific ranges of the *U*_SHE_ and pH.

Based on these findings, we selected seven *h*‐MBs (M = Sc, Ti, V, Zr, Nb, Hf, and Ta), modified with ─O and ─OH groups, for further investigation of their eCO_2_RR properties. We considered five distinct *h*‐MBenes configurations, each fully covered with either ─O or ─OH groups, and identified the most stable configuration for a detailed evaluation of their electrocatalytic performance (see Figure S5 and Tables S1 and S2, Supporting Information). We first analyzed the adsorption energies of CO_2_ on ─O terminated *h*‐MBs (referred to as *h*‐MBOs) to evaluate CO_2_ activation. The most stable CO_2_ adsorption structures on the *h*‐MBOs were identified, as illustrated in Figure S6 (Supporting Information). All adsorbed CO_2_ molecules retained a linear configuration, with minimal changes observed in both the C─O bond length and the O─C─O bond angle (Figure S7, Supporting Information). The distances between CO_2_ and the catalyst substrate ranged from 2.76 to 2.86 Å, indicating a weak interaction. The calculated adsorption energies of CO_2_ for these structures were positive, ranging from 0.26  to 0.33 eV, suggesting that spontaneous adsorption of CO_2_ is not feasible. Additionally, Bader charge analysis revealed negligible charge transfer from the *h*‐MBOs surface to CO_2_. Collectively, these findings indicate that *h*‐MBOs have limited capability to activate CO_2_, as evidenced by the positive adsorption energies and minimal changes in bond characteristics.

**Figure 4:**
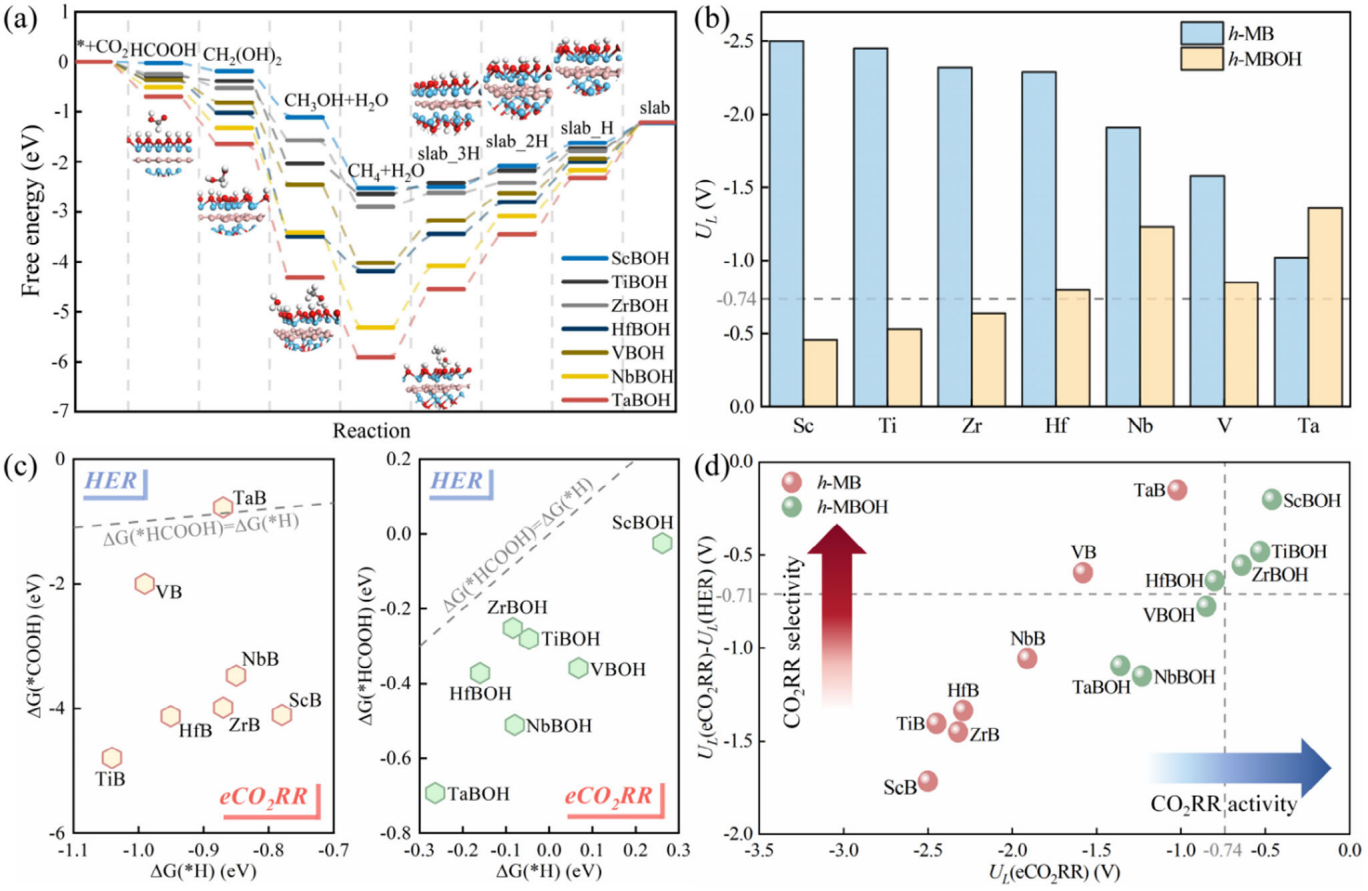
eCO_2_RR catalytic activity of bare (*h*‐MBs) and ─OH functionalized 2D *h*‐MBenes (*h*‐MBOHs). a) Calculated Gibbs free energy diagram illustrating the most feasible pathway for CO_2_ reduction to C_1_ products on *h*‐MBOHs. b) Comparison of the limiting potentials for eCO_2_RR on *h*‐MBs versus *h*‐MBOHs surfaces. c) Relationship between the Gibbs free energy change for the first hydrogenation step of eCO_2_RR and HER on *h*‐MBs and *h*‐MBOHs surfaces. d) Difference in limiting potential between eCO_2_RR and HER on *h*‐MBOHs, with the vertical dashed line indicating *U*_L_(eCO_2_RR) = −0.74 V and the horizontal dashed line representing *U*_L_(eCO_2_RR) – *U*_L_(HER) = −0.71 V.

In the interaction between CO_2_ molecules and ─OH modified *h*‐MBs (referred to as *h*‐MBOHs), it is observed that the hydrogen (H) atoms on the surface of *h*‐MBOHs combine with the carbon (C) atoms in CO_2_ to form HCOOH. However, directly obtaining the adsorption energy for this reaction is challenging. Therefore, we calculated the number of electrons transferred during the reaction of CO_2_ molecules with *h*‐MBOHs using Bader charge analysis to assess activation. The co‐acquired charges of carbon and oxygen in the generated HCOOH ranged from 0.8 to 1.0 e (Figure S8, Supporting Information). The Gibbs free energy changes for the initial reactions of *h*‐MBOHs (M = Sc, Ti, V, Zr, Nb, Hf, and Ta) are shown in **Figure** **4**a and are as follows: −2.41, −2.71, −2.86, −2.84, −3.15, −3.11, and −3.44 eV, respectively. All these values indicate that the reactions are spontaneous. These results collectively demonstrate that *h*‐MBOHs can effectively activate CO_2_ molecules.

Furthermore, we investigated the reaction pathway of eCO_2_RR on *h*‐MBOHs. We examined seven types of *h*‐MBOHs (M = Sc, Ti, V, Zr, Nb, Hf, and Ta), all of which follow the same optimal reaction pathway (Figure 4a):

(4) $$\begin{array}{ccc} {\mathrm{CO}}_{2} & \to & \mathrm{HCOOH}\to {\mathrm{CH}}_{2}{\left(\mathrm{OH}\right)}_{2}\to {\mathrm{CH}}_{3}\mathrm{OH}+H_{2}O\;\left(g\right)\\ & \to & {\mathrm{CH}}_{4}\;\left(g\right)+H_{2}O\;\left(g\right)\to \mathrm{slab}\_3H\to \mathrm{slab}\_2H\\ & \to & \mathrm{slab}\_H\to \mathrm{slab} \end{array}$$

In this pathway, “slab_*n*H” represents the presence of *n* hydrogen vacancies on the surface. Notably, the intermediates can capture hydrogen atoms from both the environment and the ─OH functional groups present on *h*‐MBOHs. The hydrogen atom bonded to oxygen originates from the proton‐electron pair in the aqueous environment, while the hydrogen atom bonded to carbon comes from the ─OH terminations of *h*‐MBOHs.

During the hydrogenation process, four hydrogen vacancies remain on the surface of *h*‐MBOHs. However, these vacancies are filled in the final four steps by acquiring hydrogen atoms from the environment, resulting in the final state being identical to the initial state. Overall, a total of eight proton‐electron pairs are transferred from CO_2_ to CH_4_. The electrocatalytic reduction of CO_2_ to CH_4_ through *h*‐MBOHs occurs spontaneously during the first four hydrogenation steps, as indicated by the negative Gibbs free energy change. Consequently, the PDS occurs during the subsequent four hydrogenation steps. The Gibbs free energy changes in these steps are all positive. Taking ScBOH as an example, the values are 0.024, 0.42, 0.46, and 0.39 eV, respectively. The PDS is identified as the transition from slab_2H to slab_H. The corresponding PDSs for the other six *h*‐MBOHs are presented in Table S3 (Supporting Information). Notably, TiBOH, ZrBOH, and HfBOH share the same PDS as ScBOH (slab_2H → slab_H), whereas the PDS for VBOH, NbBOH, and TaBOH is slab_4H → slab_3H.

By analyzing the adsorption configurations of intermediates on *h*‐MBOHs, we observe that there are no distinct activation sites on these materials. Instead, the intermediates primarily interact with the catalyst surface through hydrogen bonding. Concurrently, the catalyst surface facilitates electron transfer to the intermediates by providing hydrogen atoms. To quantify the ease with which hydrogen atoms from ─OH groups can be captured and to assess the contribution of ─OH in the electrocatalytic reduction of CO_2_, we calculated the hydrogen vacancy formation energy (*E*_Hvac_) using Equation (5):

(5) $$E_{H_{\mathrm{vac}}}=E_{\mathrm{slab}-H}\;-\left(E_{\mathrm{slab}}-E_{H}\right)$$

The calculated hydrogen vacancy formation energies (*E*_Hvac_) for the seven *h*‐MBOHs are presented in Table S4 (Supporting Information). The results indicate that the *E*_Hvac_ values for all seven *h*‐MBOHs are negative, which signifies that the release of hydrogen atoms from the ─OH functional groups is a spontaneous process. This availability of H atoms enhances the hydrogenation of the intermediates.

In addition to the reaction pathway starting with HCOOH, we also explored a possible pathway beginning with ^*^HCO_2_ (see Figure S9, Supporting Information). In this scenario, the hydrogen atom is supplied by ─OH functional groups, resulting in the formation of an H vacancy on the *h*‐MBOHs surface. As illustrated in Figure S9 (Supporting Information), during the subsequent hydrogenation of ^*^HCO_2_, regardless of whether the H atom from the environment bonds with C or O, the *h*‐MBOHs surface releases two H atoms, leading to the direct conversion into CH_2_(OH)_2_ and the generation of two new H vacancies. Following this, one O atom in CH_2_(OH)_2_ captures an H atom from the environment, while another H atom attached to this O returns to the *h*‐MBOHs surface to fill an H vacancy, ultimately leaving two H vacancies on the surface. The subsequent reaction steps mirror those of the previous pathway. Due to the strong adsorption energy of ^*^HCO_2_, the two hydrogenation processes that follow exhibit a positive Gibbs free energy change. Consequently, *h*‐MBOHs are more likely to accelerate eCO_2_RR via the first pathway starting with HCOOH.

Figure 4b summarizes and compares the limiting potentials of *h*‐MBs and *h*‐MBOHs. With the exception of TaBOH, the introduction of ─OH groups significantly enhance eCO_2_RR performance. Notably, the catalytic performances of ScBOH, TiBOH, and ZrBOH surpass that of traditional Cu catalysts (*U*_L_ = −0.74 V), with limiting potentials of −0.46, −0.53, and −0.64 V, respectively. The hydrogen evolution reaction (HER) is a significant side reaction in eCO_2_RR that can impact catalyst efficiency. To analyze the selectivity of the catalyst, we compare its performance to that of copper (Cu). The catalytic selectivity can be assessed from two angles: 1) A thermodynamic comparison of the Gibbs free energy changes for the initial hydrogenation of eCO_2_RR and HER indicates that the initiation of the reaction is more favorable for eCO_2_RR (see Figure 4c); 2) The potential difference between eCO_2_RR and HER, *U*_L_(eCO_2_RR)‐*U*_L_(HER) > −0.71 V (shown in Figure 4d), demonstrates that this catalyst exhibits better selectivity than the Cu catalyst, which has *U*_L_(eCO_2_RR) and *U*_L_(HER) values of −0.74  and −0.03 V, respectively.^[^ ^32^ ^]^ Additionally, Figure 4c reveals that for all *h*‐MBs and *h*‐MBOHs, the Gibbs free energy change for the first hydrogenation of eCO_2_RR is significantly lower than their Δ*G*(^*^H), except for TaB, which has Δ*G*(^*^COOH) = −0.77 eV, close to Δ*G*(^*^H) = −0.87 eV. This suggests that the first protonation is more likely to react with CO_2_ rather than producing ^*^H by occupying the reaction site.

To develop *h*‐MBs and *h*‐MBOHs catalysts with both high catalytic activity and selectivity, the difference in limiting potentials between eCO_2_RR and HER, *U*_L_(eCO_2_RR)‐*U*_L_(HER), for seven *h*‐MBs and seven *h*‐MBOHs catalysts is summarized in Figure 4d. The results clearly show that the eCO_2_RR pathway is favored when the *U*_L_(eCO_2_RR)‐*U*_L_(HER) value is higher. Additionally, a higher *U*_L_(eCO_2_RR) value correlates with better catalytic activity. Therefore, catalysts positioned in the upper right corner of the plot exhibit both excellent activity and selectivity, making them the most ideal candidates. In Figure 4d, the vertical dashed line indicates the catalytic activity of Cu, while the horizontal dashed line represents Cu's selectivity. Notably, ScBOH, TiBOH, and ZrBOH outperform Cu in both catalytic activity and selectivity, with ScBOH demonstrating the highest performance for eCO_2_RR.

### 2.3 Mechanism of Functional Groups Regulating eCO_2_RR Performance of *h*‐MBs

**Figure 5:**
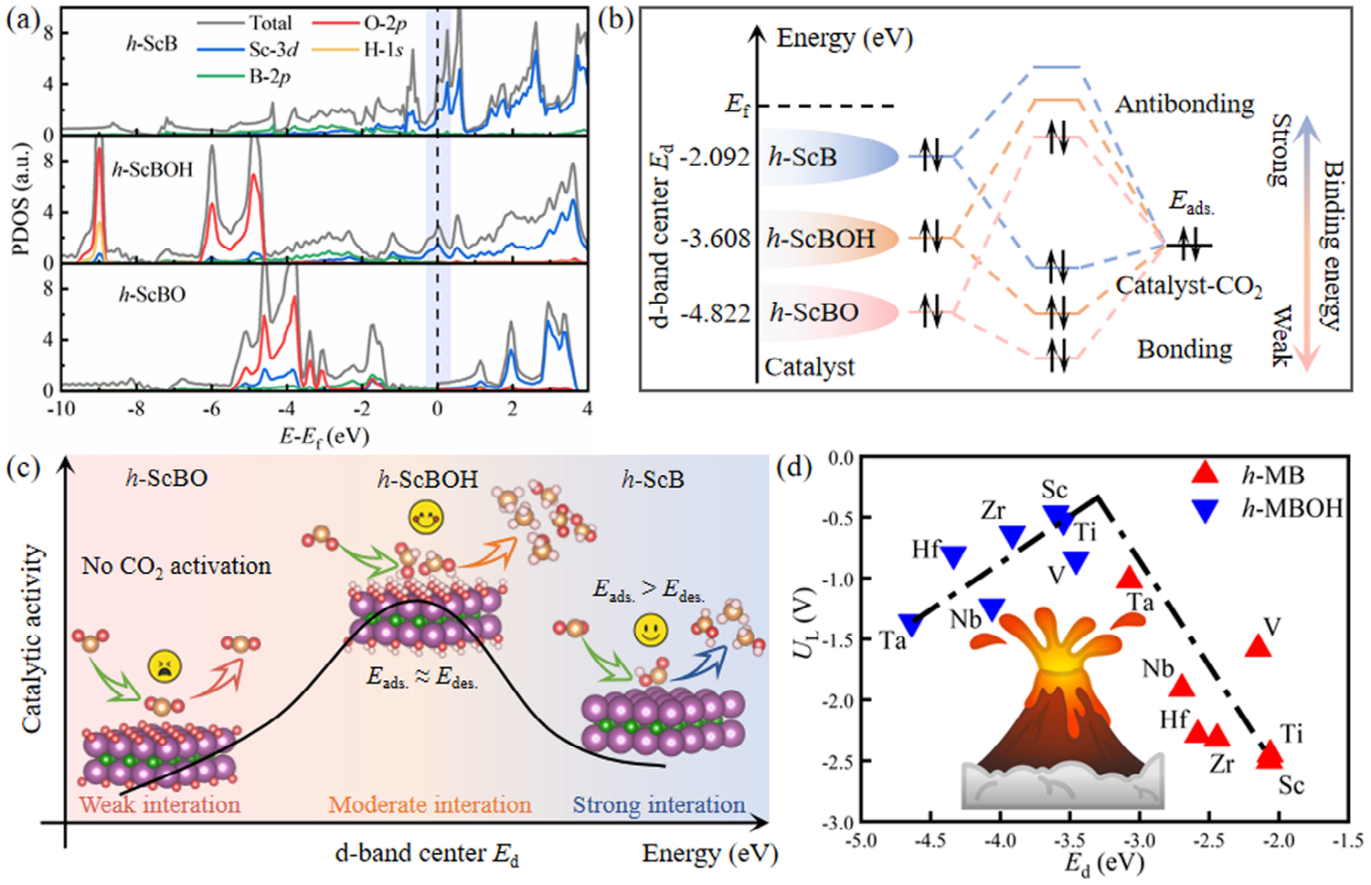
Analysis of the effect of ─O and ─OH functional groups on the eCO_2_RR catalytic performance of ScB *h*‐MB. a) Projected density of states of ScB, ScBO, and ScBOH. b) Schematic representation of the orbital interactions between the catalysts ScB, ScBO, and ScBOH with different *d*‐band centers *E*_d_ and the adsorbate CO_2_. c) Mechanism illustration of the relationship between *d*‐band center *E*_d_ and catalytic activity. d) Volcano‐shaped relationship between *U*_L_ and *E*_d._

Based on the analysis mentioned above, it is evident that functional groups have a significant impact on CO_2_ adsorption behavior. To further explore the impact of functional groups on the eCO_2_RR performance of *h*‐MBs, represented by ScB, we conducted calculations on the electronic structures of both intrinsic and functional group‐modified ScB. The projected density of states of *h*‐ScB, *h*‐ScBO, and *h*‐ScBOH are shown in **Figure** **5**a. We can note that the functional groups hybridize with the *d*‐orbitals of the transition metal, leading to the formation of new electronic states positioned significantly below the Fermi level. As shown in Figure 5a, the *d* electrons in *h*‐ScB are predominantly concentrated within the energy range of −1.6 –0 eV. In comparison, the interaction between the *d* electrons of Sc and ─OH results in the emergence of new electronic states at −8.97, −6.02, and −4.86 eV in *h*‐ScBOH. Furthermore, the hybridization of Sc's *d*‐orbitals with the *p*‐orbitals of ─O in *h*‐ScBO produces a distinct characteristic peak in the region of −6 to −4 eV. In addition, the electron transfer from Sc to ─OH or ─O increases the number of empty orbitals above the Fermi level while significantly reducing the density of states for the *d* orbitals around the Fermi level. Therefore, the incorporation of functional groups on *h*‐ScB not only alters its structural and electronic characteristics but also influences the configurations of the *d* orbital.

As illustrated in Figure 5b, when ─OH and ─O are introduced to the bare *h*‐ScB, the significant downward shifts of the *d*‐band centers (*E*_d_) relative to the Fermi level was observed in *h*‐ScBOH (−3.608 eV) and *h*‐ScBO (−4.822 eV) compared to that in bare *h*‐ScB (−2.092 eV). According to the *d*‐band theory, a downshift of *d*‐band centers can lead to an increased occupancy of the antibonding states. This phenomenon ultimately results in a weakened binding strength of CO_2_,^[^ ^33^ ^]^ which is consistent with the CO_2_ adsorption energy results. As the *d*‐band center gradually decreases, the intensity of CO_2_ adsorption follows the sequence of *h*‐ScB > *h*‐ScBOH > *h*‐ScBO. This observation clearly illustrates the essential role of functional groups in weakening the adsorption of CO_2_ on *h*‐ScB. The notable differences in adsorption behavior can be attributed to the “shielding effect” of functional groups. Specifically, the electron cloud of highly electronegative functional groups can alter the electronic structure of the matrix or eCO_2_RR intermediates.

As illustrated in Figure 5c, the adsorption of CO_2_ on *h*‐MBs is too strong, disrupting subsequent hydrogenation and product desorption processes. In contrast, CO_2_ adsorption on *h*‐MBOs is too weak to enable activation, leaving the matrix inactive as a catalyst. Therefore, to achieve optimal catalytic activity, the strength of CO_2_ adsorption must be carefully balanced. A moderate interaction between *h*‐ScBOH and adsorbates compared to *h*‐ScB and *h*‐ScBO, thereby preventing excessive intermediate adsorption or no CO_2_ activation. This mechanism enhances catalytic activity by facilitating subsequent reactions without obstruction. These findings indicate that it is feasible to introduce functional groups on *h*‐ScB to adjust the *d*‐band center and optimize the adsorption energy of CO_2_, which holds significant potential to facilitate eCO_2_RR activation and its subsequent dissociation.

The study above highlighted a strong correlation between the electronic structure—specifically the *d*‐orbitals of transition metals—and the catalytic activity in eCO_2_RR. Building on this, a volcano‐shaped relationship was established between *U*_L_ and *E*_d_, as shown in Figure 5d. It is well known that the catalytic activity for eCO_2_RR decreases as the system moves away from the peak of the volcano curve. As illustrated in Figure 5d, *h*‐MBOHs are positioned on the left side of the curve (*E*_d_ = −4.6 to −3.6 eV), while *h*‐MBs are found on the right side (*E*_d_ = −3.0 to −2.0 eV). The peak of the volcano curve occurs around −3.3 eV, with ScBOH and TiBOH near this peak, suggesting their superior catalytic activity for eCO_2_RR. This observation highlights the significant differences in the electronic structure between bare *h*‐MBs and their hydroxylated counterparts (*h*‐MBOHs), providing insights into their potential eCO_2_RR performance. These results further suggest that the introduction of functional groups to *h*‐MBs leads to a redistribution of the transition metal *d*‐orbital energy levels, which plays a crucial role in determining the eCO_2_RR catalytic performance of these materials.

### 2.4 Microscopic Kinetics Analysis

**Figure 6:**
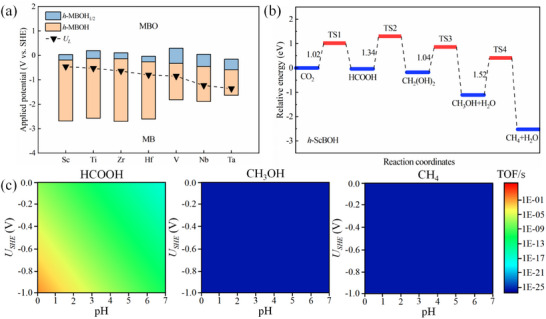
Functional group stability and microscopic kinetics analysis for the eCO_2_RR performance of ScBOH *h*‐MBene. a) Calculated applied potential for *h*‐MBenes with various surface conditions. b) Reaction pathway for eCO_2_RR on the ScBOH surface with black numbers indicating the calculated kinetic barriers for the four elementary steps leading to the formation of C_1_ products. c) Calculated turnover frequency (TOF) for HCOOH, CH_3_OH, and CH_4_.

To assess the stability of *h*‐MBOHs under the working potential (*U*_L_) of eCO_2_RR, it is necessary to confirm the functional group state on the surface of *h*‐MBOHs at the corresponding limiting potential. As shown in **Figure** **6**a, the results reveal that *h*‐MBenes terminated with ─OH groups exhibit stability under *U*_L_ during the catalytic process. Finally, the kinetics of the eCO_2_RR performance of ScBOH *h*‐MBene were further examined via microscopic kinetics method.^[^ ^34^ ^]^ Figure 6b and Figure S10 (Supporting Information) illustrate the reaction pathways on the surface of ScBOH, along with the corresponding transition state structures. Based on this kinetic analysis, the reaction pathways for C_1_ products in eCO_2_RR were identified. The energy barriers for generating HCOOH and CH_3_OH are lower than that for CH_4_ in the hydrogenation pathway. However, CH_3_OH and CH_4_ exhibit higher adsorption energies on the ScBOH surface, which limits their desorption and subsequent generation. To evaluate the relative selectivity among the products, the turnover frequency (TOF) of the three C_1_ products—HCOOH, CH_3_OH, and CH_4_—was calculated under acidic conditions (pH 0 to 7) and applied voltages ranging from 0.0 to −1.0 V. This approach helps characterize the generation rate of C_1_ products and identify optimal reaction conditions. As shown in Figure 6c, the TOF of HCOOH is significantly higher than that of the other C_1_ products under the tested conditions, peaking at a pH of 0 to 1 and requiring voltages between −0.8  and −1.0 V. Overall, this analysis indicates that HCOOH has great potential among the C_1_ products generated by eCO_2_RR, owing to its higher selectivity and lower reaction energy barrier.

## 3 Conclusion 

In summary, this study utilized DFT calculations to comprehensively evaluate the catalytic potential of 2D *h*‐MB, *h*‐MBO, and *h*‐MBOH (M = Sc, Ti, V, Zr, Nb, Hf, and Ta) for eCO_2_RR. The results reveal that MB and MBO *h*‐MBenes exhibit limited catalytic performance due to overly strong or weak interactions with reaction intermediates. However, the addition of H atoms in ─OH‐terminated MBOH *h*‐MBenes significantly enhances their catalytic activity. This improvement arises from the stabilization of reaction intermediates and a marked reduction in the limiting potential. Among the tested materials, ScBOH, TiBOH, and ZrBOH stand out as highly promising eCO_2_RR catalysts, achieving exceptionally low limiting potentials of −0.46, −0.53, and −0.64 V, respectively, surpassing the performance of metallic Cu. Electronic structure analysis further demonstrates that the introduction of ─OH groups causes a downward shift in the d‐band center of the transition metals, which moderates their interaction with adsorbed species. This optimized interaction mitigates excessive adsorption of intermediates, facilitating smoother catalytic processes. Additionally, microscopic kinetic analysis identifies HCOOH as a predominant and high‐value C_1_ product of eCO_2_RR on ScBOH *h*‐MBenes. Overall, this work provides a detailed theoretical framework for understanding the catalytic performance of *h*‐MBenes in eCO_2_RR, underscoring the transformative role of functionalization. By establishing a clear relationship between electronic structure and catalytic efficiency, this study offers valuable insights for designing advanced catalysts for sustainable CO_2_ reduction.

## 4 Computational Details

All first‐principles calculations in this work were conducted using the Vienna ab initio simulation package (VASP)^[^ ^35^ ^]^ based on density functional theory (DFT) with the projector‐augmented wave (PAW) method.^[^ ^36^ ^]^ The electron exchange‐correlation functionals were described using the generalized gradient approximation (GGA) according to the Perdew, Burke, and Ernzerhof (PBE) model.^[^ ^37^ ^]^ To minimize undesired interactions from periodic influences, a 3 × 3 × 1 supercell was constructed with a vacuum spacing of 40 Å along the *c*‐axis. A plane wave basic cutoff energy of 520 eV was selected. For geometric optimization, the Brillouin zone was sampled using a Gamma‐centred scheme with 4 × 4 × 1 *k*‐point mesh. The structure relaxation was continued until the forces acting on the system were less than |±0.05| eV Å^−1^, and the energy change was below 10^−4^/eV.

In the calculations involving electronic properties, such as the electronic density of states (DOS), a denser *k*‐point mesh of 15 × 15 × 1 was employed. To account for van der Waals interactions between layers, the DFT‐D3^[^ ^38^ ^]^ approach was utilized. Bader charge calculations, implemented in VASP, were employed to analyze atomic charge distribution. The charge density differential (CDD) was visualized using VESTA^[^ ^39^ ^]^ to illustrate charge transfer between the adsorbate and catalyst. Furthermore, transition states were identified utilizing the climbing image nudged elastic band (CI‐NEB) method,^[^ ^40^ ^]^ followed by refinement with the Dimer^[^ ^41^ ^]^ method to accurately determine the transition state structure.

The electrochemical reduction of CO_2_ to C_1_ products such as HCOOH, CH_3_OH, and CH_4_ can be represented by the following equations:

(6) $$CO_{2}+2H^{+}+2e^{-}\to \mathrm{HCOOH}+H_{2}O$$

(7) $$CO_{2}+6H^{+}+6e^{-}\to CH_{3}\mathrm{OH}+H_{2}O$$

(8) $$CO_{2}+8H^{+}+8e^{-}\to CH_{4}+2H_{2}O$$

The change in Gibbs free energy for the reaction species can be calculated using the Equation (9):

(9) ΔG=ΔE+ΔZPE−TΔS

where Δ*E* is the reaction energy calculated via DFT; Δ*ZPE* is the zero‐point energy obtained through vibrational analysis using the VASPKIT code;^[^ ^42^ ^]^ *T* is temperature (set to 300 K for this study); and Δ*S* is the change in entropy. The error in the calculated Gibbs free energies is estimated to be within 10^−3^ eV, and the reported energy values are rounded to two decimal places to reflect the precision of the calculations.

All electrochemical calculations in this study were based on the Computational Hydrogen Electrode (CHE) model.^[^ ^43^ ^]^ In this framework, the electrode potential relative to the standard hydrogen electrode (*U* vs SHE) is expressed by Equation (10)^[^ ^32^ ^]^:

(10) $$\mu \left(H^{+}\right)+\mu \;\left(e^{-}\right)=\frac{1}{2}\;\mu \left(H_{2}\right)-eU$$

For reaction A + H^+^ +  e^−^ =  B, the Gibbs free energy under the CHE model can be calculated using Equation (11):

(11) $$\begin{array}{ccc} \Delta G & = & \mu \left(B\right)-\mu \left(H^{+}\right)-\mu \left(e^{-}\right)-\mu \left(A\right)\\ & = & \mu \left(B\right)-\mu \left(A\right)-\left(\frac{1}{2}\mu \left(H_{2}\right)-eU\right) \end{array}$$

The potential determining step (PDS) is defined as the reaction step with the largest change in Gibbs free energy (Δ*G*_max_) among the *n* steps of electron transfer. Consequently, the limiting potential (*U*_L_) can be calculated as *U*_L_ = −Δ*G*_max_/*e*, where *e* represents the elementary charge (1.602 × 10^−19^ C). This limiting potential serves as an indicator for evaluating the catalytic activity of the CO_2_ reduction reaction.

To evaluate the activity and selectivity of the catalyst for eCO_2_RR, we constructed a microkinetic modeling (MKM) framework using the Catalysis Microkinetic Analysis Package (CatMAP) toolkit,^[^ ^34^ ^]^ implemented in Python. DFT‐calculated results, including free energies and transition states, were incorporated into the model, which was then used to calculate the formation rates of various reaction products. The specific calculation process of MKM has been described in detail by CatMAP, and will not be repeated here. Under fixed temperature (T = 300 K) and pressure conditions (pCO_2_ = 1 bar, pH_2_O = 1 bar), TOF values across a matrix of applied voltages (−1–0 V) and pH values (0–7) were obtained through the numerical solution of the complete reaction network (as shown in Figure 6b). The effects of voltage and pH on the formation rates of different CO_2_ reduction products were systematically visualized through 2D contour plots.

## Conflict of Interest

The authors declare no conflict of interest.

## Supporting information



Supporting Information

## Data Availability

The data that support the findings of this study are available from the corresponding author upon reasonable request.
