# Supplementary material for: Electrocatalytic CO2 Reduction Empowered by 2D Hexagonal Transition Metal Borides
Source: Adv Sci (Weinh). 2025 Apr 1;12(25):2500977. doi: 10.1002/advs.202500977 (PMC12224933; doi:10.1002/advs.202500977)
Supplement: Supplementary file 1 — Supporting Information [file ADVS-12-2500977-s001.docx]

Supporting information

Electrocatalytic CO_2_ Reduction Empowered by Two-Dimensional Hexagonal Transition Metal Borides

Yaxin Di,^+^ Zhiqi Wang,^+^ Guangqiu Wang,^+^ and Junjie Wang*

1. Di, Z. Wang, G. Wang, J. Wang

State Key Laboratory of Solidification Processing

School of Materials Science and Engineering, Northwestern Polytechnical University

Xi’an, Shaanxi 710072, People’s Republic of China

1. mail: [wang.junjie@nwpu.edu.cn](mailto:wang.junjie@nwpu.edu.cn)

Y. Di, Z. Wang, and G. Wang contribute equally to this work.

**Supplementary Figures**


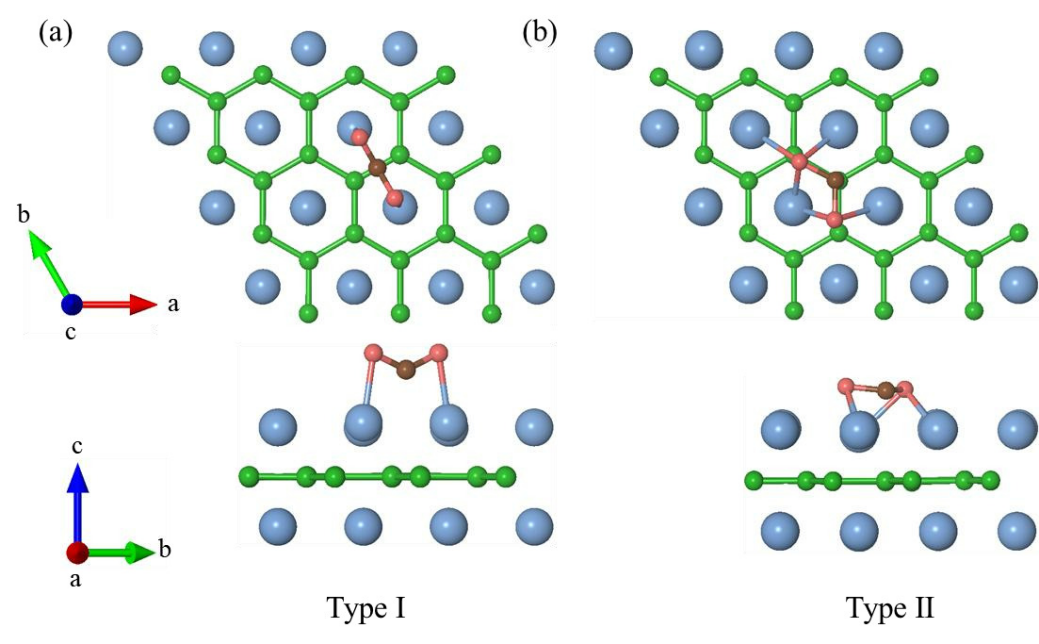


**Figure S1.** Most stable configurations for CO_2_ adsorbed on the surface of *h*-MBs: (a) Type Ⅰ for *h*-TaB and (b) Type Ⅱ for *h*-MBs (M = Sc, Ti, V, Zr, Nb, and Hf), where the green balls represent B atoms, the red balls represent O atoms, and the brown balls represent C atoms.


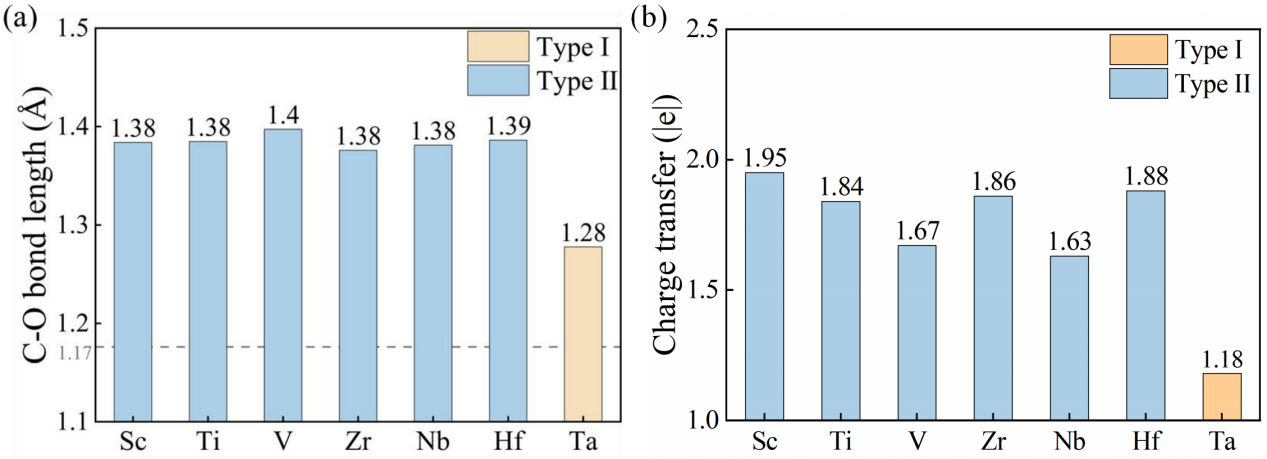


**Figure S2.** Analysis of CO_2_ activation on *h*-MBs surfaces: (a) Average value of C-O bond length in CO_2_ adsorbed on *h*-MBs and the primitive C-O bond length in CO_2_ (gray line); (b) Charge transfer between CO_2_ and *h*-MBs.
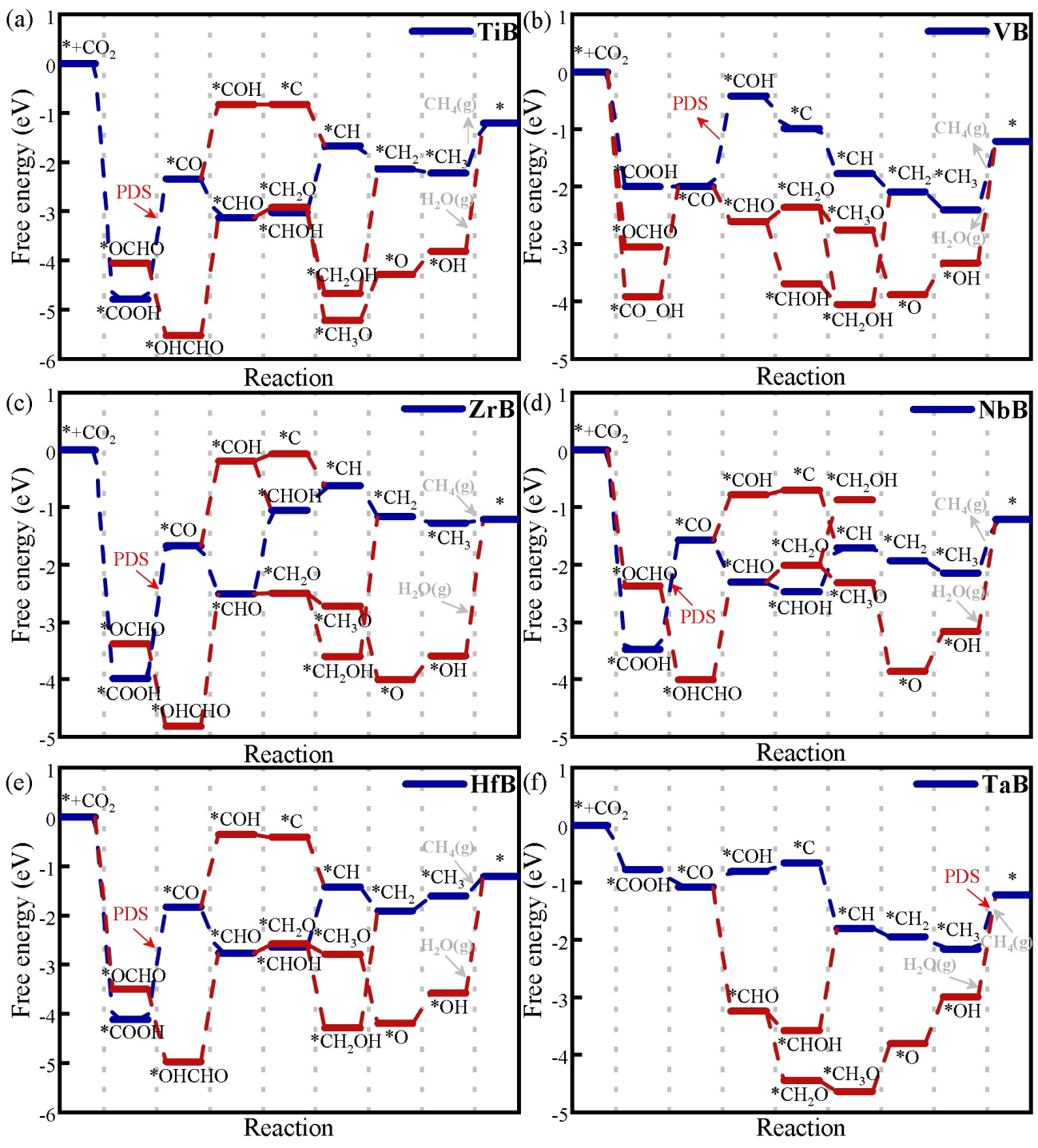


**Figure S3.** Analysis of eCO_2_RR catalytic activity on other six *h*-MBs: Calculated Gibbs free energy diagrams for eCO_2_RR pathway on (a) TiB, (b) VB, (c) ZrB, (d) NbB, (e) HfB and (f) TaB surface, where blue line represents the most favorable reaction pathway, and red line represents other possible reaction pathway.


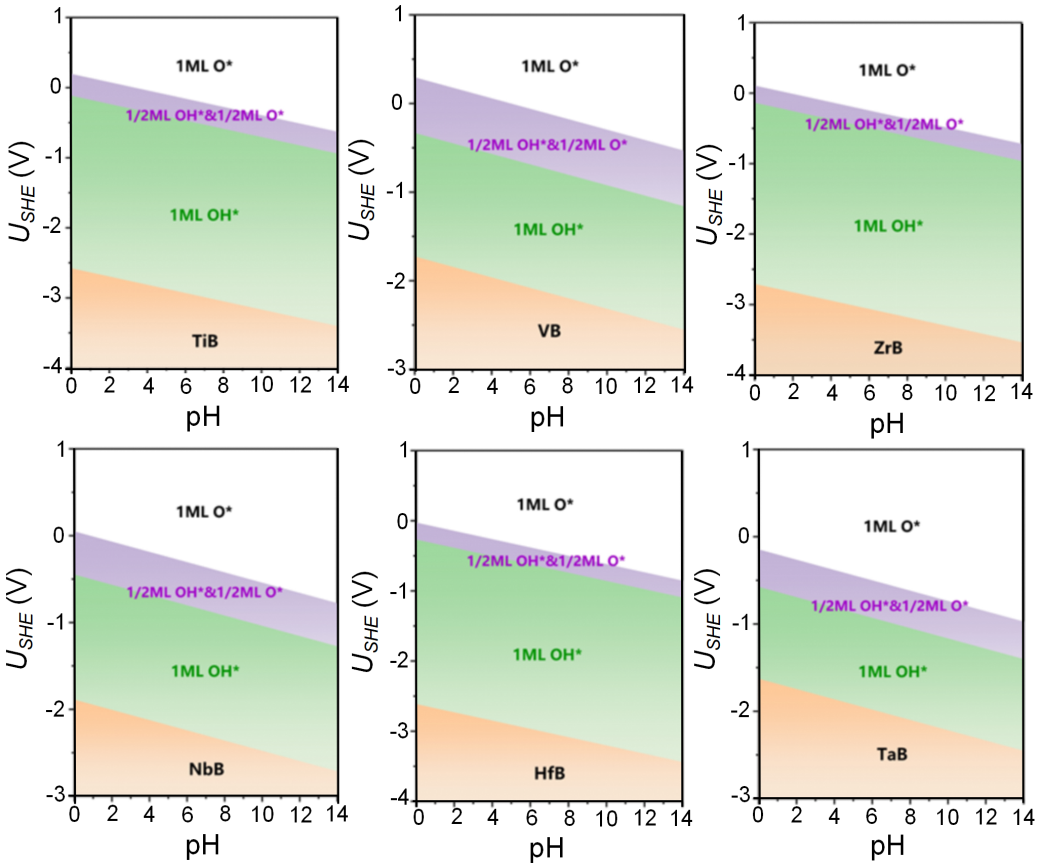


**Figure S4.** Pourbaix diagrams of other *h*-MBT*_x_* (M = Ti, V, Zr, Nb, Hf, and Ta, T = O or OH, 0 ≤ *x* ≤ 1).

As shown in **Figure S5**, in models 1 and model 2, two functional groups are located on the top of the hollow sites H1 or H2, respectively; in model 3 one functional group is located on the top of the hollow site H1, and the other one is located on the top of the hollow site H2; in model 4 both two functional groups are located on the top of the metals; in model 5, one is located on the top of metals and the other is located on the top of the hollow site.


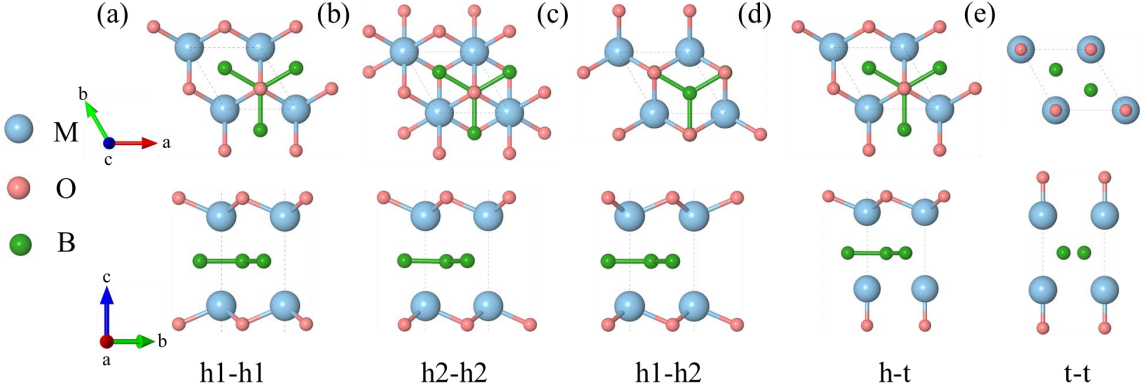


**Figure S5.** Top views (top) and side views (bottom) of five different configuration models of the -O functionalized *h*-MB: (a) h1-h1; (b) h2-h2; (c) h1-h2; (d) h-t and (e) t-t.


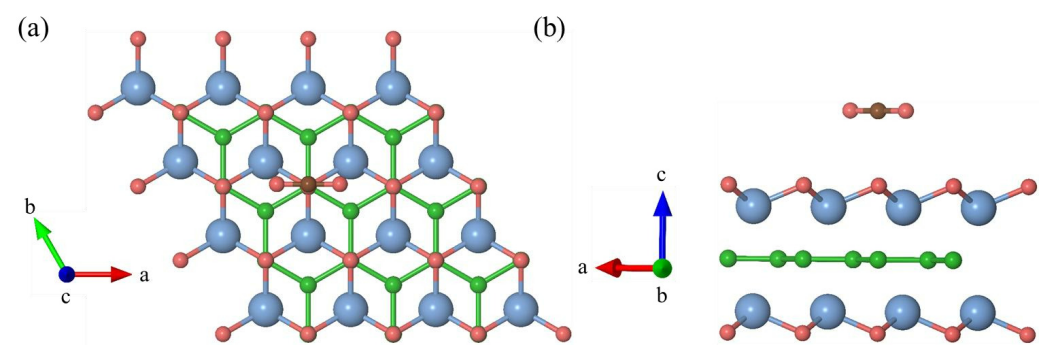


**Figure S6.** Most stable adsorption configurations of CO_2_ on the surfaces of *h*-MBOs: (a) top view and (b) side view. The red balls represent O atoms, the green balls represent B atoms, and the brown balls represent C atoms.


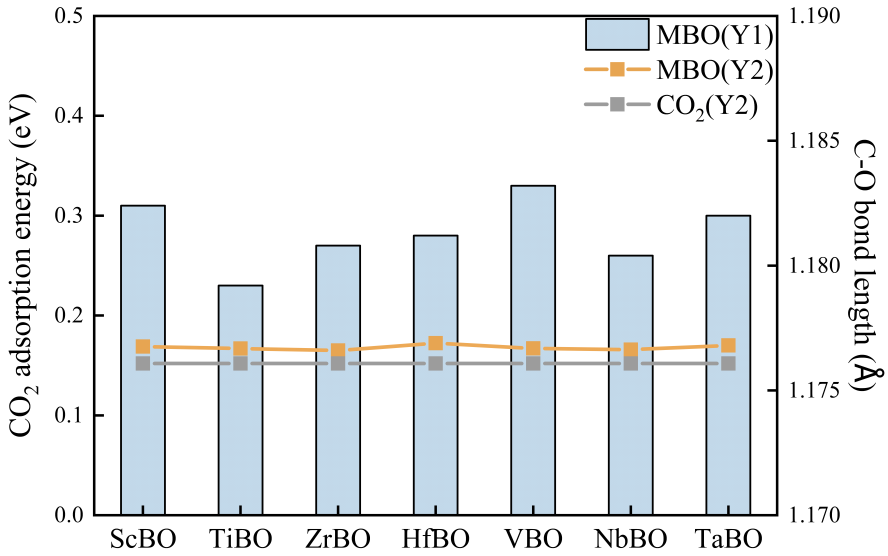


**Figure S7.** The adsorption energy of CO_2_ on *h*-MBOs (histogram), the average value of C-O bond length in CO_2_ adsorbed on *h*-MBOs (yellow line) and the primitive C-O bond length in CO_2_ (gray line).


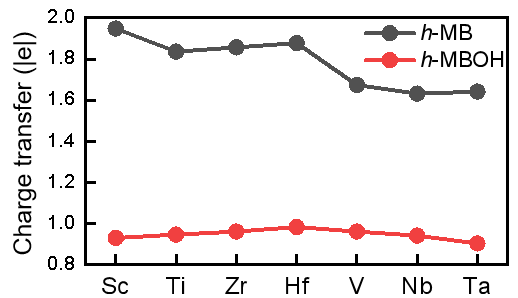


**Figure S8.** Comparison of Bader charge transfer during CO_2_ adsorption by *h*-MBs and *h*-MBOHs.


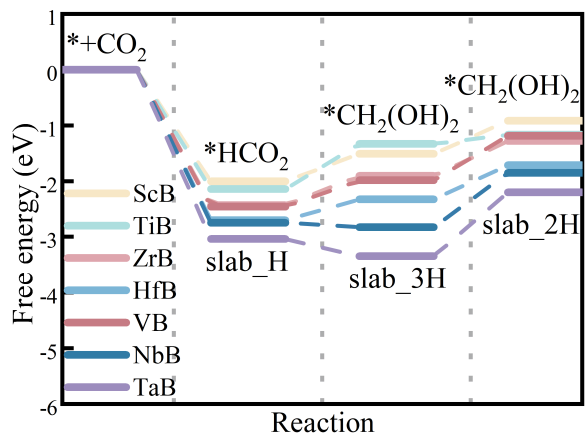


**Figure S9.** Other possible adsorbed species on the surface of *h*-MBOHs.


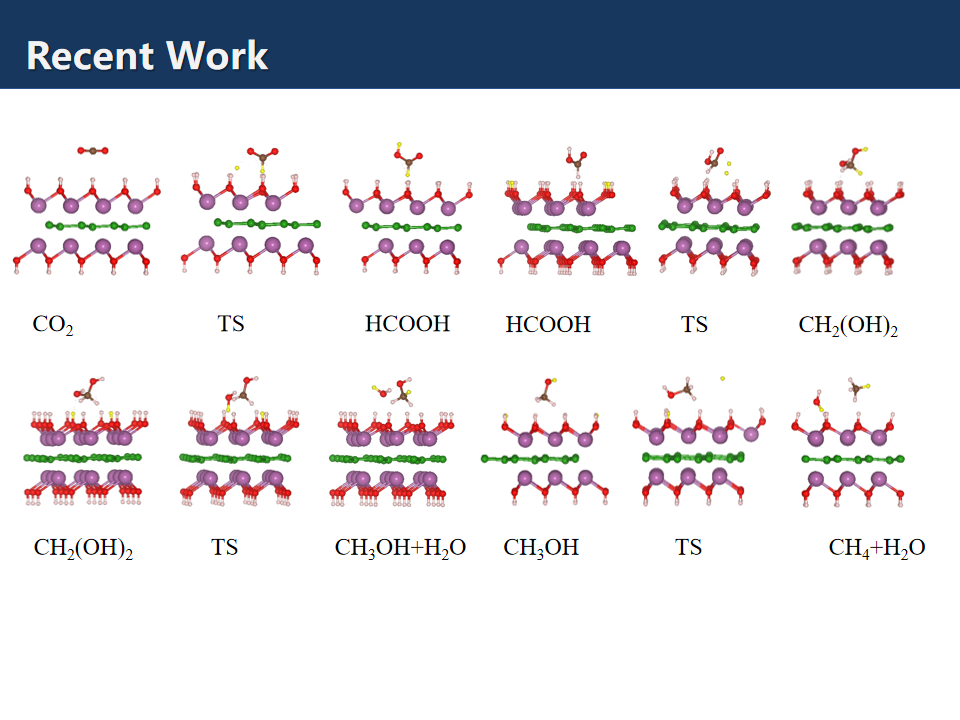


**Figure S10.** The corresponding structures for initial states, final states and transition states.

**Supplementary Table**

**Table S1** Total energy (eV) of different models for *h*-MBs (M = Sc, Ti, Zr, Hf, V, Nb, and Ta) with the surface terminations of bilayer adsorption -O

|  | h1-h1 | h2-h2 | h1-h2 | h-t | t-t |
| --- | --- | --- | --- | --- | --- |
| Sc | -48.91 | -48.92 | -48.82 | -44.41 | -40.15 |
| Ti | -51.35 | -51.35 | -51.54 | -49.18 | -46.75 |
| V | -51.14 | -51.10 | -51.02 | -49.88 | -48.70 |
| Zr | -53.03 | -53.02 | -53.24 | -50.22 | -47.19 |
| Nb | -54.83 | -54.82 | -54.59 | -52.93 | -51.15 |
| Hf | -55.96 | -55.95 | -56.24 | -52.80 | -49.41 |
| Ta | -57.86 | -57.86 | -57.62 | -55.76 | -53.80 |

**Table S2** Total energy (eV) of different models for *h*-MBs (M = Sc, Ti, Zr, Hf, V, Nb, and Ta) with the surface terminations of bilayer adsorption -OH

|  | h1-h1 | h2-h2 | h1-h2 | h-t | t-t |
| --- | --- | --- | --- | --- | --- |
| Sc | -55.28 | -55.27 | -55.33 | / | -52.42 |
| Ti | -58.32 | -58.31 | -58.24 | -57.11 | -55.99 |
| V | -57.65 | -57.64 | -57.75 | -56.94 | -56.22 |
| Zr | -59.77 | -59.76 | -59.69 | -58.51 | -57.32 |
| Nb | -60.45 | -60.46 | -60.59 | -59.77 | -58.89 |
| Hf | -62.17 | -62.16 | -62.09 | -61.02 | -59.93 |
| Ta | -62.82 | -62.81 | -62.97 | -62.33 | -61.78 |

**Table S3** Potential determining step of eCO_2_RR on *h*-MBOHs surface

|  | ScBOH | TiBOH | VBOH | ZrBOH | NbBOH | HfBOH | TaBOH |
| --- | --- | --- | --- | --- | --- | --- | --- |
| PDS | slab_2H→slab_H | slab_2H→slab_H | slab_4H→slab_3H | slab_2H→slab_H | slab_4H→slab_3H | slab_2H→slab_H | slab_4H→slab_3H |

**Table S4** Hydrogen vacancy formation energy of *h*-MBOHs

|  | ScBOH | TiBOH | VBOH | ZrBOH | NbBOH | HfBOH | TaBOH |
| --- | --- | --- | --- | --- | --- | --- | --- |
| *E_Hvac_* (eV) | -0.39 | -0.53 | -0.72 | -0.57 | -0.96 | -0.79 | -1.11 |
